# T140. RESTING STATE NETWORKS ALTERATION IN SCHIZOPHRENIA

**DOI:** 10.1093/schbul/sby016.416

**Published:** 2018-04-01

**Authors:** Gianluca Mingoia, Igor Nenadic

**Affiliations:** 1 RWTH Aachen University; 2 Jena University Hospital

## Abstract

**Background:**

While functional MRI and PET studies have shown altered task-related brain activity in schizophrenia, recent studies suggest that such differences might also be found in the resting state (RS). Here we used ICA based analysis to investigate RS fMRI data to compare connectivity of 11 well known networks (Auditory, Cerebellum, DMN, Exectutive Control, Fronto-parietal 1, Fronto-parietal 2, Salience, Sensorimotor, Visual1, Visual2, Visual3 network) between patients with schizophrenia and healthy controls suggesting deficits in related neuropsychological functions.

**Methods:**

We obtained RS fMRI series (3T, 3x3x3mm resolution, 45 slices, TR 2.55s, 210 volumes) in 25 schizophrenia patients (mean age 30a±7.3), on stable antipsychotic medication and 25 matched healthy controls (30.3a±8.6). Subjects were asked to lie in the scanner keeping eyes closed with no further specific instructions. Data were pre-processed; we applied FSL MELODIC (pICA) yielding IC, we used FIX to auto-classify ICA components which represent artifacts and an automated routine to select for each subject the component matching the anatomical definition of resting state networks. SPM12 was used for second level analysis, we used two sample t-test to compare networks functional connectivity between groups. We then analysed the power spectrum density (PSD) for the extracted networks, estimating the power of the signal at different frequencies.

**Results:**

Our method reliably identified all networks in every control and patients. We found significant differences in the anatomical pattern of areas. Patients showed decreased functional connectivity in comparison to healthy controls in portions of Cerebellum, DMN, Executive Control, Fronto-parietal1 and sensorimotor networks; in addition patients showed increased functional connectivity in comparison to healthy controls in portions of DMN network. Finally, PSD was found altered in patients with Schizophrenia when compared to healthy controls in Fronto-parietal1, Sensorimotor, visual1and visual2 networks.

**Discussion:**

Well known resting state networks were reliable identified from RS fMRI in Schizophrenia patients. The differences in anatomical distribution point to possible alterations in functional connectivity in Schizophrenia, which suggests disruption in Cerebellum, DMN, Executive control, Fronto-parietal and sensorimotor activity. The comparison of the PSD suggests changes in Fronto-parietal1, Sensorimotor and in addition visual1, visual2 networks.

